# Life History Responses and Gene Expression Profiles of the Nematode *Pristionchus pacificus* Cultured on *Cryptococcus* Yeasts

**DOI:** 10.1371/journal.pone.0164881

**Published:** 2016-10-14

**Authors:** Gaurav V. Sanghvi, Praveen Baskaran, Waltraud Röseler, Bogdan Sieriebriennikov, Christian Rödelsperger, Ralf J. Sommer

**Affiliations:** Max Planck Institute for Developmental Biology, Department of Evolutionary Biology, Spemannstraße 37, Tübingen, Germany; University of Massachusetts Medical School, UNITED STATES

## Abstract

Nematodes, the earth’s most abundant metazoa are found in all ecosystems. In order to survive in diverse environments, they have evolved distinct feeding strategies and they can use different food sources. While some nematodes are specialists, including parasites of plants and animals, others such as *Pristionchus pacificus* are omnivorous feeders, which can live on a diet of bacteria, protozoans, fungi or yeast. In the wild, *P*. *pacificus* is often found in a necromenic association with beetles and is known to be able to feed on a variety of microbes as well as on nematode prey. However, in laboratory studies *Escherichia coli* OP50 has been used as standard food source, similar to investigations in *Caenorhabditis elegans* and it is unclear to what extent this biases the obtained results and how relevant findings are in real nature. To gain first insight into the variation in traits induced by a non-bacterial food source, we study *Pristionchus*-fungi interactions under laboratory conditions. After screening different yeast strains, we were able to maintain *P*. *pacificus* for at least 50–60 generations on *Cryptococcus albidus* and *Cryptococcus curvatus*. We describe life history traits of *P*. *pacificus* on both yeast strains, including developmental timing, survival and brood size. Despite a slight developmental delay and problems to digest yeast cells, which are both reflected at a transcriptomic level, all analyses support the potential of *Cryptococcus* strains as food source for *P*. *pacificus*. In summary, our work establishes two *Cryptococcus* strains as alternative food source for *P*. *pacificus* and shows change in various developmental, physiological and morphological traits, including the transcriptomic profiles.

## Introduction

The phylum Nematoda (roundworms) is one of the most diverse groups of animals, whose members occupy almost every ecological niche on earth [1]. Their numerical abundance and omnipresence, and the existence of a diversity of life cycles point towards an important role of nematodes in many ecosystems with functions at various trophic levels [2]. As a result, nematodes show an enormous range of feeding strategies [3] with many of the estimated 1–10 million species being specialists that cannot be cultured under laboratory conditions. In contrast, some nematode species are easily cultured using *Escherichia coli* or other bacteria as food and have been developed as model organisms in basic biology, the best studied example being *Caenorhabditis elegans* (www.wormbook.org). Similarly, its distant cousin *Pristionchus pacificus* has been established as a second laboratory model with similar molecular and genetic tools available [4, 5].

*P*. *pacificus* is a self-fertilizing hermaphrodite and belongs to the family Diplogastridae [6]. It has first been established as a satellite model organism in evolutionary developmental biology (evo-devo) due to several technical features that allow functional and mechanistic studies under laboratory conditions. These technical features include i) a short generation time of four days at 20°C, ii) a large brood size, and iii) the ability to be cryopreserved [7]. Further, an annotated genome, whole genome re-sequencing data of 104 *P*. *pacificus* strains and the mapping of the pharyngeal connectome have complemented large-scale efforts in developmental genetics based on forward and reverse genetic tools, all of which allowed detailed comparisons of developmental processes between *P*. *pacificus* and *C*. *elegans* [8–12]. More recently, *P*. *pacificus* has been developed as a model organism for integrative studies in evolutionary biology that try to link laboratory-based, mechanistic studies with field work in ecology and population genetics [13]. In the wild, *P*. *pacificus* is often found in association with scarab beetles in an interaction that is described as entomophilic or necromenic [14,15]. Necromeny is the phenomenon where a nematode associates with a living insect or other invertebrate in the growth-arrested dauer stage, an alternative, third larval stage [16]. After the death of the vector, nematodes resume development and feed on microbes, including bacteria, fungi, protozoans, which come together to decompose the carcass.

Additionally, *P*. *pacificus* has more recently been used as a model to study phenotypic plasticity, the ability of a genotype to produce distinct phenotypes in different environmental conditions [17]. Specifically, *P*. *pacificus* shows a mouth dimorphism and individual animals irreversibly develop either a narrow-mouthed “stenostomatous” (St) or a wide-mouthed “eurystomatous” (Eu) form [18]. Eu animals have two teeth allowing predatory feeding on other nematodes, whereas St animals with only one tooth are strict microbial feeders. *P*. *pacificus* mouth-form development is controlled by various conditional (i.e. starvation, crowding) and stochastic factors, making *P*. *pacificus* a model system to study the genetic, molecular and environmental control of phenotypic plasticity [17] For example, detailed studies revealed the involvement of small molecules in mouth-form development and they identified the sulfatase-encoding *eud-1* gene as a developmental switch controlling Eu *vs*. St development [19, 20].

Microbial communities present in ecological niches play a critical role in nematode ecology, behavior and physiology. However, while behavioral, neurobiological and physiological studies for food selection were reported in many animal species, like rats (*Rattus norvegicus*), mink (*Mustela vison*), moth caterpillars (e.g. *Heliothis zea*) and predatory beetles (*Agonum dorsale*)[21–24], little is known about omnivorous nematodes and their behavioral and physiological responses to different food sources. Only recent studies in *C*. *elegans* have started to investigate the influence of different bacteria and yeast on this model organisms physiology [25, 26]. For *P*. *pacificus*, beetle-associated bacteria [27, 28] have been isolated, but they have largely been characterized with regard to their potential pathogenicity. Furthermore, yeast or other fungi have never been investigated as potential food source for *P*. *pacificus*.

Given that most research on *C*. *elegans* and *P*. *pacificus* is performed in the background of *E*. *coli* as food, it is unclear to what extent this precondition influences the investigated traits and how important the underlying molecular mechanisms are in controlling a given trait in real nature. In this study we ask the question, how variable various life-history traits and transcriptomic profiles are between worms that are grown on different food sources. We chose yeast strains for comparison because we anticipated that a comparison with a non-bacterial organism would maximize the observed differences relative to *E*. *coli* and would be the best starting point to estimate the overall magnitude of expected variation when comparing different bacteria as food sources. We therefore first tried to find yeast strains of the *Cryptococcus* genus, on which *P*. *pacificus* can complete its life cycle for multiple generations and characterize life history traits, such as development time, brood size, mouth-form ratio, and defecation time of *P*. *pacificus* in order to confirm that these traits are comparable to worms that are grown on *E*. *coli*. Furthermore, we measured gene expression profiles of *P*. *pacificus* grown on *Cryptococcus* to estimate the amount of transcriptomic changes on different food sources and to find novel candidates for previously un-described genes that might be important under specific environmental conditions.

## Materials and Methods

### Nematode and yeast strains

*P*. *pacificus* PS312 was grown and maintained on nematode growing media (NGM) plates seeded with *E*. *coli* OP50 at 20°C. Different *Cryptococcus* yeast strains viz. *C*. *flavus*, *C*. *humicola*, *C*. *curvatus*, *C*. *tephrensis* and *C*. *terreus* were purchased from Deutsche Sammlung von Mikroorganismen und Zellkulturen (DSMZ), Braunschweig, Germany. Yeast strains were maintained on Potato dextrose agar media (Sigma-Aldrich Chemie GmbH, Germany) and on media 186 as prescribed by DSMZ for culture maintenance of some yeast strains. All yeast strains were maintained at 30°C. In order to minimize differences for media composition, pH and temperature, the activated broth cultures of yeast were seeded on NGM media similar to *E*. *coli* OP50.

### Selection of yeast strains suitable for culturing *P*. *pacificus*

Among the tested strains, the two yeast strains *C*. *albidus* (C3) and *C*. *curvatus* (C5) were facilitating the growth of *P*. *pacificus*. Furthermore, both the strains form transparent lawns on NGM facilitating the observations for life span assays. *P*. *pacificus* strains were cultivated on *Cryptococcus* strains for multiple generations (~ 50–60 generations). These two strains were used for further experimentation. To avoid any bacterial contamination, unseeded plates were inoculated with a mixture of chloramphenicol and streptomycin antibiotic solutions. The same procedure was done for control OP50 plates.

### Life history and physiological traits of yeast-fed worms

#### 1. Development Time

For studying developmental time, 20–50 young adults were transferred to agar plates seeded with yeast at 20°C. Eggs hatched within 1–2 hours from young adults were transferred to new plates and observed till new eggs of the next generation appeared after self-fertilization. Eggs were transferred by a metal pick to new plates. In all protocols, NGM media plates with *E*. *coli* OP50 were kept as control and the same procedure was followed for *E*. *coli* OP50 at 20°C. Ten plates were used per replicate and experiments were repeated two times.

#### 2. Survival and brood size assay

Yeast cultures were activated in Potato dextrose broth by overnight culture in a shaking incubator at 30°C. 500 μl of cultures were seeded on 6 cm NGM plates and incubated overnight. Assays were performed with 20 J4 *P*. *pacificus* animals at 20°C. Survival was monitored for 10 days. After every two days, *P*. *pacificus* was transferred onto fresh yeast plates. Individuals, which did not respond to a touch by a metal pick were considered dead. For brood size, 10 J4 larvae were transferred to agar plates with yeast or *E*. *coli* OP50. Hatched progeny were counted as J2 or J3 larvae.

#### 3. Mouth-form phenotyping

For mouth-form phenotyping, *P*. *pacificus* was grown for four generations on both yeasts as well as *E*. *coli* OP50 plates in order to exclude transgenerational effects induced by the transfer. Hundred individuals were randomly picked from plates to score Eu and St mouth-form frequencies. The mouth-form was observed by Differential Interference Contrast microscopy (DIC) and screened in five biological replicates as previously described [29].

#### 4. Defecation time and Pharyngeal pumping rate

For defecation assays, overnight-activated liquid cultures were seeded on NGM plates and incubated overnight. Nematodes feeding on yeast and *E*. *coli* OP50 were starved for five hours on NGM plates. Starved worms were transferred on yeast and OP50 containing plates. Plates were not disturbed for 15–20 minutes so that nematode could adjust to the culture medium. Defecation rate was defined as the number of defecation recorded in a 10-minute time interval by a single nematode. Defecation was observed using a Carl Zeiss Discovery microscope (Germany). In order to examine the nature of yeast clumps in the nematode intestine, 10 adults feeding on yeast cultures were photographed using DIC microscope. To verify that altered defecation rate was not linked to permanent attachment of potentially pathogenic yeast cells to the walls of intestinal lumen, the same individuals were put on empty plates and were allowed to defecate for 15–20 minutes. After that, nematodes were removed and plates were incubated for two days to test if yeast cells could still grow after passing through digestive system of the worm.

For counting pharyngeal pumping rate, 10 young adults were transferred on 10 seeded yeast strain plates. Worms were kept undisturbed for 10 min for recovery and initiating regular pumping. Pharyngeal pumping rates were observed via the Zeiss Axio imager a1 microscope for 15 seconds under both *Cryptococcus* species and *E*. *coli* OP50 strains. For each worm, pumps were counted for 15 seconds and then multiplied its mean by four to derive the mean pumps per minute.

### Statistical Analysis

Mouth-form phenotyping, brood size, chemotaxis scores, defecation time were compared using student t-test for means. The survival of *P*. *pacificus* (10 days) grown on *Cryptococcus* species was analyzed using Kaplan–Meier tests. For testing for differences in developmental timing (egg to egg), brood size, pumping rate, defecation rate, and mouth-form differences, we applied Kruskal-Wallis test with posthoc chi-squared tests.

### Gene expression profiling

For gene expression profiling, adult worms were picked and frozen at -80°C. Frozen worms were homogenized by multiple freeze—thaw cycles. Total RNA was isolated using standard Trizol extraction following the manufacturers’ instructions (Ambion, CA, USA). RNA concentration was quantified using Qubit and Nanodrop measurements (Invitrogen Life technologies, CA, USA). RNAseq libraries were prepared using TruSeq RNA library preparation kit v2 (Illumina Inc, CA, USA) according to the manufacturer’s instructions from 1 μg of total RNA in each sample. Libraries were quantified using Qubit and Bioanalyzer measurements (Agilent Technologies, CA, USA) and normalized to 2.5 nM. Samples were sequenced as 150bp paired end reads in one multiplexed lane using the HiSeq2000 platform (Illumina Inc, CA, USA).

### Analysis of RNA-seq data

Raw reads were aligned to the *P*. *pacificus* reference genome (version Hybrid1) using TopHat (version 2.0.14) [30]. We downloaded the TAU2011 annotation of *P*. *pacificus* genes from pristionchus.org and estimated expression levels for each data set separately using cufflinks (version 2.0.1) [31]. Principal component analysis was done using R. Differential expression analysis was done using cuffdiff (version 2.0.1) [31]. For all programs, default parameters were used. Sets of differentially expressed genes were compared with previous expression profiling studies [32–34] and enrichment was tested using Fisher's exact test. Similarly metabolic pathway annotations for *C*. *elegans* were downloaded from http://www.genome.jp and *P*. *pacificus* genes were annotated with a given pathway if they fell into an orthologous cluster as defined by the orthoMCL software [35].

## Results

After screening several *Cryptococcus* strains for their potential to serve as alternative food source for *P*. *pacificus*, we were able to maintain nematode cultures for multiple generations (~ 50–60 generations) on the two yeast strains *C*. *albidus* (C3) and *C*. *curvatus* (C5). Furthermore, both strains form transparent lawns on NGM, which facilitates the observation in life span assays. We therefore decided to use these two strains for further experiments.

### Developmental Timing

**Fig 1:**
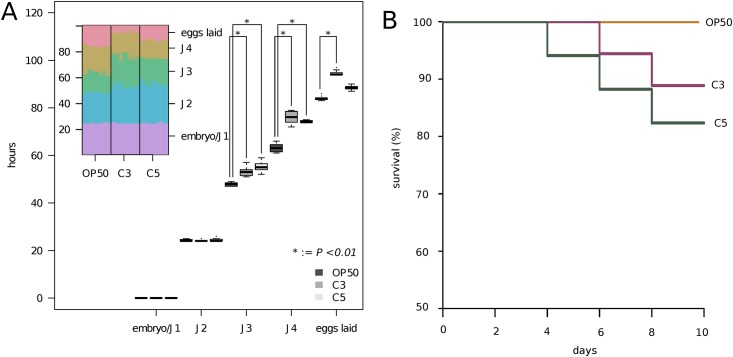
Developmental timing and survival curves of *P*. *pacificus* after exposure to *C*. *albidus* and *C*. *curvatus*. A) The boxplots show the time needed for *P*. *pacificus* nematodes to enter different developmental stage when growing on *E*. *coli* OP50 or *Cryptococcus* strains. The inlay shows the individual data points representing ten plates per food source. B) Survival of *P*. *pacificus* exposed to *C*. *albidus* (C3) and *C*. *curvatus* (C5) for 10 days. Standard lab food *E*. *coli* OP50 was used as a control.

Food source is known to be one of the primary factors influencing generation time (from egg to egg) in nematodes. We compared development rate of *P*. *pacificus* cultured on *E*. *coli* OP50, *C*. *albidus* (C3) and *C*. *curvatus* (C5) (Fig 1A). We found no significant differences in the speed of embryonic development between nematodes grown on OP50 and both yeast strains (Fig 1A). However, significant differences (P<0.01) were observed during larval development (Fig 1A). Specifically, starting from the J3 larval stage, *P*. *pacificus* grew slower on both yeast strains than on OP50 (Fig 1A).

#### Survival and Brood size assay

We assessed survival of *P*. *pacificus* on both *Cryptococcus* strains. On *C*. *albidus*, a more than 90% survival rate was observed after eight days of incubation, close to the survival seen for *E*. *coli*-fed worms. Survival on th *C*. *curvatus* was somewhat lower, but still 80% of animals were alive after 10 days of incubation (Fig 1B). These data demonstrate that all yeast-fed worms are alive at the onset of reproduction and a sufficient proportion of individuals survive during the early reproductive period, which permits indefinite propagation of *P*. *pacificus* on *C*. *albidus* and *C*. *curvatus*.

**Fig 2:**
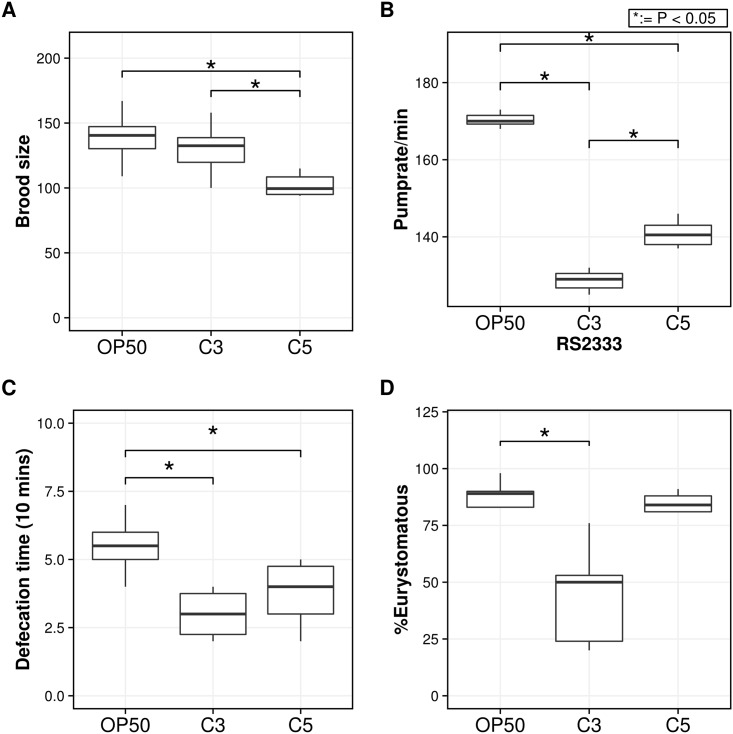
Life history traits of *P*. *pacificus* cultured on the yeasts *C*. *albidus* and C. *curvatus*. The boxplots show the median, first and third quartile values from different life history traits experiments. A) Brood size of *P*. *pacificus* grown on *C*. *albidus* (C3), *C*. *curvatus* (C5) and *E*. *coli* OP50. Brood size was calculated as average number of progeny of 10 J4 larvae. B) Pharyngeal pumping rate results of *P*. *pacificus* exposed to *Cryptococcus* species. Assay was performed using *E*. *coli* OP50 as control. C) Defecation time (10 mins) of *P*. *pacificus* exposed to *C*. *albidus* (C3) and *C*. *curvatus* (C5) using *E*. *coli* OP50 as control. D) Eurystomatous (Eu) ratio of *P*. *pacificus* adults grown on *C*. *albidus* (C3) and *C*. *curvatus* (C5). Eu ratio was calculated from five biological replicates. Statistical significance was calculated using student t-test.

Brood size measurements revealed similar differences between *C*. *albidus* and *C*. *curvatus*, and *E*. *coli* OP50 (Fig 2A). Specifically, self-fertilizing hermaphrodites laid 140 (median) eggs on *E*. *coli* OP50 seeded plates and 130 (median) eggs were laid on *C*. *albidus* plates. In contrast, on *C*. *curvatus* plates only 100 (median) eggs were observed. This number is significantly different in comparison to the number of eggs laid on *E*.*coli* OP50 and *C*. *albidus* (P<0.05) (Fig 2A). These results suggest that *C*. *albidus* can be considered to represent a comparable food source for maintaining the reproductive life cycle of *P*. *pacificus*. In contrast, culturing *P*. *pacificus* on *C*. *curvatus* results in reduced survival rates and brood sizes.

#### Pharyngeal puming rate and Defecation Assays

Pharyngeal pumping rate was observed via Zeiss Axio imager a1 microscope for 15 seconds for both *Cryptococcus* and *E*. *coli* OP50. When *P*. *pacificus* was fed on *E*. *coli* OP50 the median pumping rate was 170 compared to median pumping rate of 70 on *C*. *albidus* and 140 on *C*. *curvatus*. Thus, a significant difference in the mean (P<0.05) was observed on both yeast strains when compared to *E*. *coli* OP50 (Fig 2B).

**Fig 3:**
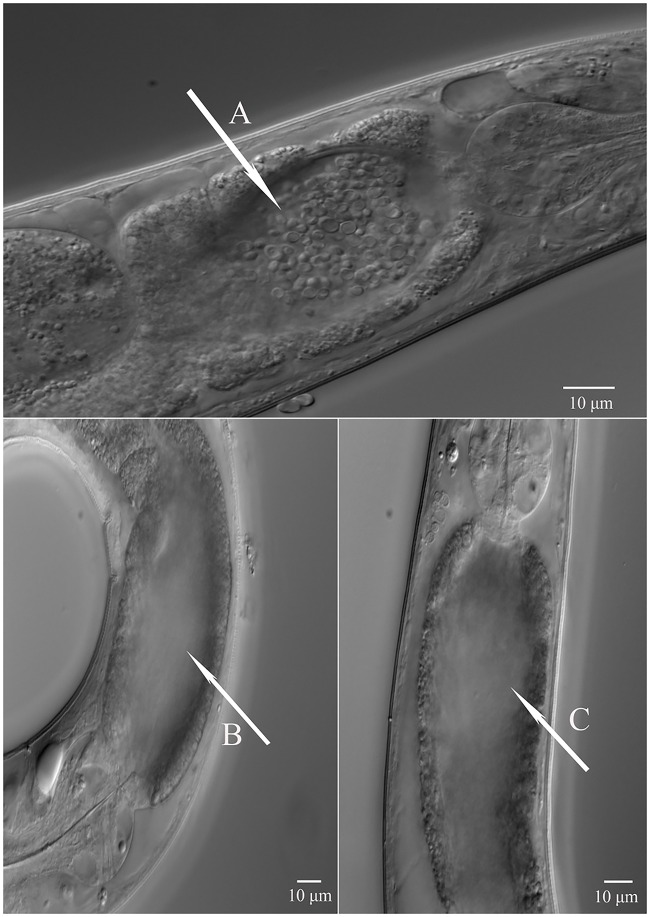
Differential Interference Contrast microscopy (DIC) to study nature of yeast clumps. A) Yeast cells clumps of *C*. *albidus (C3)* in intestine of *P*. *pacificus*. B), C) Absence of yeast clumps in defecated worms after 3 hours off food, suggesting that yeasts cells can be fully digested or excreted by *P*. *pacificus*.

When *P*. *pacificus* was fed on *E*. *coli* OP50 the defecation rate (10 min) was 5.5 compared to 3 on *C*. *albidus* and 4 on *C*. *curvatus*. Thus, a significant difference in the mean defecation rate (P<0.05) was observed on both yeast strains when compared to *E*. *coli* OP50 (Fig 2C). Also, clumps of yeast cells were observed in the intestine of the nematodes. To test if these clumps were due to indigestibility of yeast cells, yeast-feeding nematodes were transferred to unseeded NGM plates and were allowed to defecate. After three hours on unseeded plates, yeast clumps were no longer present in the intestine of worms suggesting that yeasts cells can be digested or excreted by *P*. *pacificus*. Furthermore, these findings suggest that the larger cell size slows down defecation time (Fig 3).

#### Mouth-form plasticity

Next, we tested the influence of continuous culturing of *P*. *pacificus* on *C*. *albidus* and *C*. *curvatus* on the mouth-form ratio. On *E*. *coli* OP50, the *P*. *pacificus* reference strain RS2333 has a 90–70%Eu:10–30%St ratio [17]. In the culturing conditions used in our experiments, we observed a Eu mouth-form frequency of median 89% (Fig 2D). In contrast, worms grown on *C*. *albidus* and *C*. *curvatus* formed 50% and 84% Eu animals, respectively (Fig 2D). Thus, feeding *P*. *pacificus* on *C*. *albidus* increases the St mouth-form frequency (P<0.05).

#### Yeast diet induces substantial transcriptomic responses

**Fig 4:**
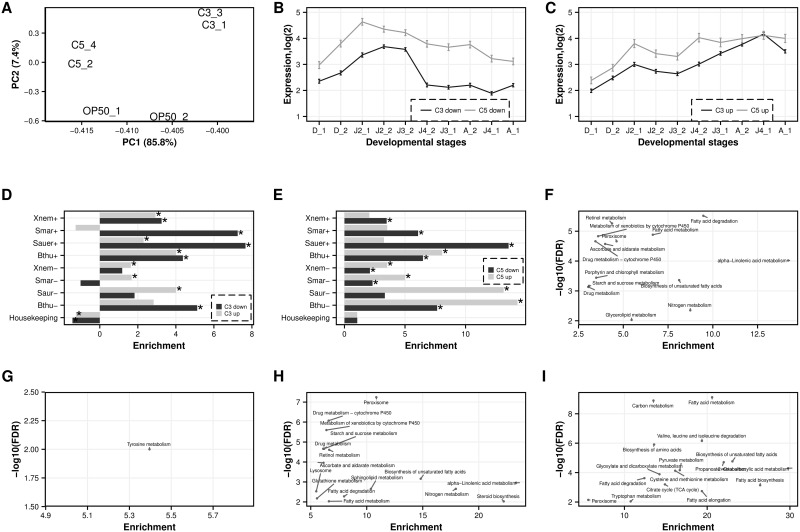
Gene expressions profiling of *P*. *pacificus* after a *Cryptoccocus* or *E*. *coli* diet. A) PCA of transcriptomes obtained from *P*. *pacificus* adult worms growing on *C*. *albidus* (C3), *C*. *curvatus* (C5) and *E*. *coli* OP50. Despite substantial variation in the control samples grown on *E*. *coli* OP50, different samples cluster together according to their food source indicating robust expression changes under different environments. B) Genes that are down-regulated upon *Cryptococcus* diet show a bias towards higher expression at early developmental stages. The graph shows mean expression and standard error of down-regulated genes in 10 developmental transcriptomes representing dauer, J2, J3, J4, and adult worms. C) Genes that are up regulated upon *Cryptococcus* tend to reflect transcriptomes from later developmental stages. D) Enrichment of significantly differentially expressed genes upon exposure to *C*. *albidus (C3)* in previous gene expression profiling studies. E) Enrichment of significantly differentially expressed genes upon exposure to *C*. *curvatus (C5)* in previous gene expression profiling studies. F) KEGG pathway enrichment of down-regulated genes under exposure to *C*. *albidus (C3)*. The x-axis shows the enrichment score indicating how much more genes of a given pathway are found as differentially expressed when compared to random gene sets. The y-axis shows the negative logarithm of the p-value indicating the significance of the overrepresentation. G) KEGG pathway enrichment of up-regulated genes under exposure to *C*. *albidus* (C3). H) Enrichment of KEGG pathways among down-regulated genes when fed with *C*. *curvatus* (C5). I) Enrichment of KEGG pathways among up-regulated genes after exposure to *C*. *curvatus* (C5).

The experiments described above show that a *Cryptococcus* diet influences several physiological characteristics of *P*. *pacificus* relative to an *E*. *coli* diet. To study the influence of diet on gene expression profiles, we sequenced the transcriptomes of hand picked young adult *P*. *pacificus* worms that were grown on the two yeast strains and used worms grown on *E*. *coli* OP50 as reference. For each strain, two biological replicates were sequenced resulting in a total of 49–70 million reads (2x150bp). Despite substantial variation between the two control samples grown on *E*. *coli*, principal component analysis of expression values shows a clear separation between transcriptome profiles of worms grown on different food sources (Fig 4A). We identified 716 (319 up, 397 down) genes significantly differentially expressed in response to exposure to *C*. *curvatus* (FDR corrected P<0.05). In contrast, 2518 (1431 up, 1087 down) genes were differentially expressed in response to exposure to *C*. *albidus* (C3).

To characterize the identified gene sets in greater detail, we compared our findings with results from previous gene expression profiling studies in *P*. *pacificus*. Interestingly, despite the fact that only adult worms were picked for RNA extraction, in both transcriptomes significantly differentially expressed genes exhibited a strong overlap with genes, previously identified as developmentally regulated [33]. More precisely, 786 (57%, P<10^−250^) of genes that are up-regulated in response to exposure to *C*. *albidu*s were previously described as developmentally regulated. Similarly 168 (53% P<10^−50^) of up-regulated genes in response to *C*. *curvatus* are also developmentally regulated. To illustrate these trends, we plotted the mean expression level of all significantly differentially expressed genes (yeasts *vs*. *E*. *coli*) across the developmental transcriptomesshowing that up regulation in response to both yeast strains seem to correlate with expression at later developmental stages (Fig 4B and 4C) [33]. Similarly, genes that are up-regulated in response to *E*. *coli* are biased towards transcriptomes of earlier developmental stages. This finding might suggest that genes whose expression is food source dependent are in addition also developmentally regulated. Alternatively, it is also possible that the observed pattern is a secondary effect of slower development (Fig 1A).

#### Housekeeping functions can still be maintained

Based on the comparison of the differentially expressed genes to previous gene expression profiling studies in *P*. *pacificus* [32–34], we found that exposure to both yeast strains only showed a mild effect on housekeeping functions (Fig 4D and 4E). These findings are clearly different from the response to bacterial pathogens, such as *Serratia marcescens* and *Xenorhabdus nematophila* that kill *P*. *pacificus* within five days [32], which is reflected in a massive breakdown of housekeeping function at the transcriptomic level [33]. These findings suggest that both yeast strains are not toxic or pathogenic to *P*. *pacificus*. Moreover, known pathogen-induced genes show a tendency to be significantly down regulated when worms are grown on the both yeast strains as opposed to *E*. *coli* OP50 (Fig 4D and 4E).

#### General activation of protein, sugar, and fatty acid metabolism

We complemented the characterization of the transcriptomes by overrepresentation analysis of gene families (as defined by PFAM domains) and metabolic pathways (KEGG). Similar to the analysis of previous expression profiling studies, we found that in response to both yeasts, Cytochrome P450 genes, which are associated with the detoxification of xenobiotics, are significantly down regulated in response to both yeast strains (Fig 4F and 4H). Again, this observation could potentially suggest that *E*. *col*i OP50 is actually more toxic than *Cryptococcu*s. In addition, genes up-regulated in response to exposure to *C*. *curvatus* (Fig 4I) showed enrichments in protein ('Biosynthesis of amino acids', P<10^−7^, Fisher's exact test), sugar ('Gylcoslyis/Glucogenesis', P<10^−2^), and fatty acid metabolism ('Fatty acid metabolism', P<10^−2^). The only significantly enriched pathway for *C*. *albidus* was 'Tyrosine metabolism' (Fig 4G). Given that both yeast strains lead to a developmental delay, these results could potentially suggest that yeast cells are either more difficult to digest or are less nutritious. We speculate that worms have to invest more energy in synthesize essential molecules and therefore, develop slower.

## Discussion

Several studies in the recent past have investigated the tritrophic interactions of bacteria, beetles and *Pristionchus* nematodes [27], whereas the potential interaction of *Pristionchus* with fungi or yeast was never specifically investigated under *in vitro* conditions. Therefore, the present study is the first of its kind and aims to investigate the interaction of yeast with *Pristionchus* by investigating the effects of *C*. *albidus* and *C*. *curvatus* on life history traits and gene expression profiles of *P*. *pacificus*. Our study results in four major conclusions.

First, *P*. *pacificus* is able to grow and reproduce on *C*. *albidus* and *C*. *curvatus* yeast strains. Despite the relatively large size of yeast cells, *P*. *pacificus* was able to complete its life cycle in 3.9 days and 3.7 days on *C*. *albidus* and *C*. *curvatus* at 20°C, respectively. These values are comparable to the generation time of 3.5 days on *E*. *coli* OP50. In addition, our data on survival and brood size of *P*. *pacificus* on both yeast strains indicate that *P*. *pacificus* can survive and finish its self-fertilizing reproductive cycle when feeding on *C*. *albidus* or *C*. *curvatus* although the latter reduces survival and brood size.

Second, we found a significant change in mouth-form ratios of *P*. *pacificus* when fed on *C*. *albidus*. Specifically, 50% Eu animals were observed on *C*. *albidus*, whereas 89% Eu animals were found on *E*. *coli* (Fig 3D). This change in mouth-form frequency might be influenced by several factors. For example, the relatively large size and density of yeast cells will influence nematode metabolism and energy consumption, which eventually may lead to a metabolic cost of plasticity. We speculate that the on average faster larval development of St animals [29] is favored when the nematode metabolizes yeast cells. The gene expression profiles of *P*. *pacificus* cultures on yeast cells suggest that young adults are more similar to later adult stages grown on *E*. *coli* OP50. This finding (see below for a more detailed discussion) would support a potential metabolic cost hypothesis. However, it should be noted that it is inherently difficult to estimate metabolic and other costs for plastic traits [36].

Third, survival, brood size and generation time assays, nor the defecation studies showed extreme effects of both *Cryptococcus* strains. While effects of *C*. *albidus* and/or *C*. *curvatus* on various life history traits were observed, none of them resulted in growth arrest or a heavily increased mortality. The precise fitness effect was strongest for *C*. *curvatus* reducing brood size. However, it should be noted that in the wild, nematodes are not exposed to monoxenic cultures of microbes as those used in laboratory studies. Consistently, nematodes recovered from decomposed beetle carcasses usually have a diversity of microbes in their intestine [27](Meyer and Sommer, manuscript in preparation).

Finally, our analysis of transcriptomic changes in response to yeast exposure overlapped significantly (P<10^−250^) with gene sets that were previously known as developmentally regulated [33]. Currently it is unclear, whether this strong association is truly a yeast-induced response or represents a secondary effect of slower development. If the latter explanation were correct, this would suggest that the transcriptomic age of a worm might not necessarily correspond to its morphological stage. However, to decide on this question requires much more detailed expression profiling studies. The transcriptomic signal of the remaining genes basically support the two previous findings as general housekeeping functions can still be maintained and pathogen related genes tend to be down-regulated.

In conclusion, the present study establishes two *Cryptococcus* strains as alternative food source for *P*. *pacificus* and shows for the first time in a systematic manner the interactions of *P*. *pacificus* with yeast. We found that *Pristionchus* was able to continue its life cycle on two yeast strains and had developmental time, brood size and survival rate comparable to worms grown on the common lab food *E*. *coli* OP50. *Pristionchus* shows considerable mouth form dimorphism when fed on yeasts indicating an important role of feeding plasticity when *Pristionchus* is exposed to different food sources in nature. Future studies should aim to understand the genetic regulation involved in different food choices.
